# Clinical features of the initial cases of 2009 pandemic influenza A (H1N1) virus infection in an university hospital of Morocco

**DOI:** 10.1186/1755-7682-3-26

**Published:** 2010-10-27

**Authors:** Maha Louriz, Chafiq Mahraoui, Abderrahim Azzouzi, Mohamed Taoufiq El Fassy Fihri, Amine Ali Zeggwagh, Khalid Abidi, Driss Ferhati, Salma Echcherif El Kettani, Rajae Tachinante, Jihane Belayachi, Aicha Zekraoui, Yasser Sefiani, Al Mountacer Charif Chefchaouni, Redouane Abouqal

**Affiliations:** 1 Ibn Sina University Hospital, 10000, Rabat, Morocco; 2 Faculté de Medecine et de Pharmacie-Université Mohamed V, 10000, Rabat, Morocco

## Abstract

**Background:**

The first case of 2009 pandemic influenza A (H1N1) virus infection in our center was documented on June 15. Subsequently, persons with suspected cases of infection and contacts of those with suspected infection were tested. Persons in whom infection was confirmed were hospitalized and quarantined, and some of them were closely observed for the purpose of investigating the nature and duration of the disease. The aim of the present study was to describe baseline characteristics, treatment, outcomes, hospital length of stay and mortality of the first 186 cases of influenza A (H1N1) virus infection, with special interest in those developing severe respiratory failure with intensive care unit (ICU) care requirement.

**Methods:**

observational study of 186 consecutive cases of influenza A (H1N1) virus infection admitted in 3 departments that were reference centers for the care of patients with influenza A and 4 ICU in Ibn Sina university hospital (Rabat, Morocco) between June and December 2009. Real time reverse-transcriptase-polymerase-chain-reaction (RT-PCR) testing was used to confirm infection. Demographic data, symptoms, comorbid conditions, illness progression, laboratory and chest radiologic findings, treatments, clinical outcomes and ICU care requirement were closely monitored.

**Results:**

The mean age of the 186 patients was 17.6 ± 14.8 years, 47.8% had less than 14 years and 57% were male. The median duration of symptoms before hospital admission was 3 days (interquartile range (IQR): 2-5). The most common symptoms were fever (in 91.5% of the patients), cough in 92.5%, and nasal congestion in 62.4%. Twenty four percent of patients had comorbid respiratory disorders and 7.5% were pregnant. Abnormalities in chest radiography were detected in 26.3% of 186 patients on admission or after hospitalization. Twenty patients have required ICU care and 10 have required mechanical ventilation. The hospital length of stay was 5 days (IQR: 4-5). The following were risk factors of ICU admission: older age (p = 0.03), long duration of symptoms (p = 0.07), asthma (p = 0.01), obesity (P < 0.001), abnormalities of chest radiography (P < 0.001), leukocytosis (p = 0.005), and higher C-reactive protein (CRP) (P < 0.001). The ICU length of stay was 4 days (IQR: 3-6.7). The mortality rate was 3.5% among all patients and 30% among ICU patients.

**Conclusions:**

Close observation of patients infected with the 2009 pandemic influenza A (H1N1) virus infection provided us with several information. The influenza A (H1N1) virus infection affected young people particularly, with comorbid respiratory disorders. Risk factors of ICU admission were older age, long duration of symptoms, asthma, obesity, abnormalities of chest radiography, leukocytosis and higher CRP. Clinicians should be aware of complications of influenza A (H1N1) virus infection, particulary in patients with risk factors.

## Background

In April 2009, cases of human infection with 2009 pandemic influenza A (H1N1) virus were identified in the United States and Mexico [1,2] and then the virus spread rapidly to other regions of the world [3,4]. Thus, the world health organization (WHO) raised the pandemic level from 5 to 6, the highest level [5]. The 2009 H1N1 virus is a triple resorting influenza virus containing genes from human, swine and avion influenza viruses [6-8]. The transmission human to human of the virus was documented [9]. Influenza pandemics of the past century have been associated with a remarkably consistent epidemiologic curve, with peaks in the spring, fall, and later winter [10]. The first case of confirmed infection with the virus in our center was documented on June 15, 2009. Since this was a new and potentially serious infection disease, all patients with confirmed infection who had been hospitalized were quarantined in the hospital to isolate them from the general population. To our Knowledge, there are no studies that investigated influenza A (H1N1) virus infection in developing countries. Some patients with influenza A (H1N1) virus infection have developed rapidly progressive lower respiratory tract disease resulting in respiratory failure with attributed death. Morocco has a total population of 31,285,174; gross national income per capita is $ 3.860. The health budget corresponds to 1.1 percent of gross domestic product and 5.5 percent of the central government budget. Morocco has inadequate numbers of physicians (0.5 per 1,000 people) and hospital beds (1.0 per 1,000 people) and poor access to water (82% of the population) and sanitation (75% of the population). The health care system includes 122 hospitals; 2,400 health centers; and 4 university clinics, but they are poorly maintained with inadequate capacity to meet the demand for medical care. Only 24,000 beds are available for 6 million patients seeking care each year, including 3 million emergency cases [11]. Morocco has two major health sectors, public and private, said to be complementary rather than competitive. Patients may choose whether to attend primary or secondary, public or private care. The majority of Moroccans in employment pay for health insurance, which covers most, but not all, of health expenses within the public and private sector. This report summarizes the clinical characteristics of a series of the first 186 patients reported to be admitted to our center due to influenza A (H1N1) virus infection in Morocco, with special interest in those developing severe respiratory failure.

## Methods

### Study design and setting

We retrospectively studied all patients with confirmed influenza A (H1N1) admitted between June and December 2009, to 3 departments that were reference centers for the care of patients with influenza A (Medical emergency department, paediatric department of infectious diseases and gynaecology-obstetric department) and 4 intensive care units (ICUs) (Medical ICU, surgical ICU, paediatric ICU, and gynaecology-obstetric ICU) in Ibn Sina university hospital of Rabat, Morocco. Data abstracted for this study were obtained from a voluntary registry institute by all departments that were reference centers after the first known (H1N1) influenza A cases.

### Data collection

Data were reported by attending physicians reviewing medical charts, radiologic and laboratory records. Inclusion criteria consisted of febrile (≥38°C) acute illness, respiratory symptoms consistent with cough, sore throat, nasal congestion, myalgia, acute respiratory failure requiring ICU admission, a history of travel to a country where infection had been reported in the previous 7 days or an epidemiologic link to a person with confirmed or suspected infection in the previous 7 days plus microbiologic confirmation of novel influenza A (H1N1) virus. Persons suspected of being infected and persons identified as close contacts were quarantined for 5 days, during which time pharyngeal or nasopharyngeal swabs were collected for detection of the virus by means of real-time reverse-transcriptase-polymerase-chain-reaction (RT-PCR). The average time between obtaining the samples and testing was 2 hours. A confirmed case was defined by a positive result of a RT-PCR assay performed at a reference laboratory in Ibn Sina Hospital. Only confirmed cases were included in the current study. Person with confirmed cases of infection were admitted to different departments, where they could be quarantined. The ICU admission criteria and treatments decisions for all patients including determination of the need for intubation and type of antibiotic and antiviral therapy administered were not standardized and were made by the attending physicians. We defined critically ill patients as those admitted to an adult or paediatric intensive care unit, requiring mechanical ventilation, or receiving intravenous infusion of inotropic or vasopressor medication during the hospitalization. Patients were followed until discharge with symptoms and signs recorded daily. The details of all investigations and treatments were recorded. The criteria for discharge were two readings of normal body temperature taken on 2 consecutive days and the absence of respiratory symptoms. Patient's confidentiality was maintained and this study was approved by the local ethics committee. The following informations were recorded: demographic data, country of origin of the contagion, symptoms, comorbidities , time off illness onset, chest radiologic, laboratory and microbiologic findings, ICU admission, mechanical ventilation requirement, need for vasopressor drugs, antiviral treatment, adverse event during hospital stay, length of stay in the hospital, length of stay in the ICU, and the hospital mortality.

### Statistical analysis

Data analysis was conducted using SPSS 13.0 software (Chicago, IL, USA). Data represented as the mean ± standard deviation for variable with a normal distribution, and as the median with interquartile range (IQR) for variable with skewed distribution. For categorical variables, the percentages of patients in each category were calculated. Clinical, biological and radiologic characteristics were compared between subgroups of patient with and those without ICU admission. Differences between subgroups were assessed using the Chi-squared test and Fisher's exact test for categorical variables and the Student's T-test or Mann-Whitney test for continuous variables. A *P *value of 0.05 or less was considered to be statistically significant.

## Results

### Demographic and clinical characteristics of the patients

Three departments located in 3 hospitals were involved in the health response. The first case of confirmed imported infection in our center was identified on June 15, 2009; and the first secondary case of confirmed infection (in a patient with known exposure to a person with imported infection) was identified on July 8. By December 2009, a total of 872 close contacts and suspect cases had been tested for 2009 pandemic influenza A during quarantine, test results were positive for 186 persons (21.3%). We analysed the first 186 cases with confirmed cases of infection who were hospitalized. Forty patients had travelled to infected region (table 1) (21.5%). The median duration of symptoms before the hospital admission was 3 days (IQR: 2-5). Fever (91.5%) and cough (92.5%) were more common in our patients. Of all patients, 50.5% had underlying medical conditions. Asthma was more common in our patients (24%). Demographic details, underlying medical conditions and symptoms are listed in table 1. Table 2 showed characteristics of ICU patients with 2009 pandemic influenza A (H1N1) virus.

**Table 1:** Characteristics, underling medical conditions and symptoms of 186 patients infected with 2009 pandemic influenza A (H1N1) virus in Morocco.

Characteristics	Values
**Male gender**, n (%)	106 (57)
**Age **(yrs)	
Mean ± SD	17.6 ± 14.8
Range	0.08-57
**Age group**, n (%)	
<5 yrs	53 (28.6)
5-14 yrs	37 (20)
15-30 yrs	55 (29.2)
31-50 yrs	38 (20.5)
>51 yrs	3 (1.7)
**Coexisting conditions**, n (%)	
Asthma	48 (24)
Pregnancy	15 (7.5)
Heart disease	13 (6.5)
Haematologic disease	10 (5)
Chronic nephritis	4 (2)
Diabetes	2 (1)
Obesity	2 (1)
**Recent travel to infected region**, n (%)	40 (21.5)
None	146 (78.5)
Spain	9 (22.5)
France	8 (20)
Canada/USA	7 (17.5)
United Kingdom	4 (10)
Italy	3 (7.5)
Others	9 (22.5)
**Duration of symptoms **(days)	
Median (IQR)	3 (2-5)
**Symptoms**	
Elevated temperature (>38°C)	170 (91.5)
Cough	172 (92.5)
Sore throat	52 (28)
Nasal congestion	116 (62.4)
Myalgia, arthralgia	78 (41.9)
Diarrhea	26 (14)
Nausea, vomiting	30 (16.1)
Headache	56 (30.1)
**Hospital length of stay **(days)	
Median (IQR)	5 (4-5)

IQR, interquartile range; SD, standard deviation; yrs, years; USA, United States of America.

**Table 2:** Characteristics of ICU patients infected with 2009 pandemic influenza A (H1N1) virus.

Characteristics	Values
**Age**, years	
Mean ± SD	25.9 ± 18.8
Range	0.25-53
**Male gender**, n (%)	12 (60)
**Asthma**, n (%)	6 (22.2)
**Obesity**, n (%)	2 (7.4)
**Pregnancy**, n (%)	3 (11.1)
**Length of stay**, days	
Median (IQR)	4 (3-6.7)
**Mortality**, n (%)	6 (30)

IQR, interquartile range; SD, standard deviation.

### Laboratory and radiographic findings

Table 3 showed laboratory and radiographic findings on admission. We had 20 requirements for ICU care (10.7%), and 10 requirements for mechanical ventilation (5.3%). The results of serial virologic testing of samples from pharyngeal or nasopharyngeal swabs with the use of RT-PCR assay were available for all our patients. Abnormalities in chest radiography were detected in 49 cases on admission or after hospitalization. The most common features of these abnormalities appeared as ground-glass opacities. Leukocytosis was found in 50.9%.

**Table 3:** Laboratory and radiographic findings in admission.

Variables	Values
**Abnormalities on chest radiograph**, n/total n (%)	49/186 (26.3)
Opacity 1/4 quadrants	24/49 (48.9)
Opacity 2/4 quadrants	19/49 (37.7)
Opacity 3/4 quadrants	4/49 (8.1)
Opacity 4/4 quadrants	2/49 (4)
**Leukocyte count**	
Median (IQR) (per mm3)	14600 (5400-16100)
<4000/mm^3^, n/total n (%)	4/102 (3.9)
>10000/mm^3^, n/total n (%)	52/102 (50.9)
**Platelet count**,	
Mean ± SD (per mm3)	221241 ± 169344
**Potassium**	
Median (IQR) (meq/liter)	4.9 (3.6-5.1)
<3.5 meq/liter, n/total n (%)	14/61 (23)
**Sodium**, mean ± SD (meq/liter)	136 ± 5.2
**C-reactive protein **>10 mg/liter, n/total n (%)	55/72 (76.3)
**Creatinine**, median (IQR) (mg/dl)	8.9 (7.4-11.3)
**Aspartate aminotransferase **>40 UI/liter, n/total n (%)	10/30 (33.3)
**Alanine aminotransferase **>40 UI/liter, n/total n (%)	10/30 (33.3)

IQR, interquartile range; SD, standard deviation.

### Clinical outcomes

96.5% of our patients were discharged home after a hospital length of stay of 5 days (IQR: 4- 5). Treatment with Oseltamivir was administered in a total of 184 patients within 48 hours after the onset of illness. Twenty patients required ICU care and 10 required mechanical ventilation. The following were risk factors of ICU admission (table 4): older age (p = 0.03), long duration of symptoms (p = 0.07), asthma (p = 0.01), obesity (P < 0.001), abnormalities of chest radiography (P < 0.001), leukocytosis (p = 0.005), and higher C-reactive protein (CRP) (P < 0.001). The hospital mortality was 3.5% in our study. The mortality among ICU patients was 30%.

**Table 4:** Risk factors of ICU admission among patients infected with 2009 pandemic influenza A (H1N1) virus.

Variables	Ward	ICU	P. value*
**Age**, year (mean ± SD)	14.3 ± 10.8	26.5 ± 19.6	0.030
**Male sex**, n (%)	94 (56.6)	12 (60)	0.700
**Duration of symptoms**, days (median (IQR))	2 (2-4)	4 (2-7)	0.070
**Asthma**, n (%)	41 (24.6)	6 (30)	0.010
**Obesity **, n (%)	0 (0)	2 (10)	<0.001
**Pregnancy**, n (%)	12 (7.2)	3 (15)	0.200
**Abnormalities on chest radiography**, n (%)	34 (20.5)	15 (75%)	<0.001
**Leukocyte count**, per mm^3 ^(mean count ± SD)	8670 ± 1450	13741 ± 1027	0.005
**C-reactive protein**, mg/liter (mean ± SD)	23.1 ± 16.2	138.6 ± 102.7	<0.001

* *P *values are from the Chi-squared test, Student's T-test or Mann-Whitney test; IQR, interquartile range; SD, standard deviation.

## Discussion

We describe a cohort of 186 patients identified in Ibn Sina university hospital who were hospitalized for 2009 pandemic influenza A (H1N1) virus infection between June and December 2009. The decision to undertake stringent quarantine and isolation measures regarding these patients was based on the unknown biologic action of the virus and the absence of effective vaccination. Several studies suggested an incubation period of 1 to 7 days [6,9]. In our study, we couldn't provide the opportunity to investigate the incubation period without an exact date of onset of illness. To date, it has been difficult to define the true incubation period of the virus [6,9,12,13]. As compared with patients in United States [6], Japan [14], and other countries, the fever was the main symptom (94%), the same result was found in our study (91.5%). The incidence of nausea, vomiting and diarrhea was much lower than previously reported. Both leukocytosis and leukopenia were found in our cohort as well as other studies [15]. As compared with patients in China [9], fewer patients in our cohort presented leukopenia (white-cell count, <4000 per cubic millimetre) (3.9% vs 21.4%). These transient alterations in the numbers of peripheral-blood leukocytes are similar to those seen in cases of seasonal influenza. Fas-Fas ligand signalling, which induces apoptosis, plays a major role in the mechanisms regulating the leukocyte population [16]. Hypokaliemia was documented in 23% of our patients. Hypokaliemia was found in 25.4% in China [9]. The Excessive loss by the gastrointestinal tract (diarrhea, and vomiting), appear to be the most obvious reason that potassium levels are low. However, nausea and vomiting can be a cause or a consequence of hypokalemia. As compared with patients in United State [6] and china [9], abnormalities on chest radiography were more common in our cohort (26.3% vs 5.1% in china, 7.1 in USA). Twenty patients had severe pneumonia requiring ICU care. Our analysis of critically ill patients with 2009 influenza A (H1N1) reveals that this disease affected an older patients group. Other study revealed that H1N1 infection affected young patients [17]. There was a relatively long period of illness prior to presentation to the hospital among critically ill patients. The median time between the onset of symptoms and ICU admission was 2 (2-4) vs 4 (2-7) in ward. Late management of the disease may increase the severity of H1N1 infection, as it is suggested by other studies [18].

Asthma and obesity were the most common comorbid conditions in critically ill patients. Obesity and respiratory diseases were more prevalent in others studies especially in critically ill patients with H1N1 infection [17]. A better understanding of these factors, which were common, or those that suggest a higher risk of ICU admission may provide health care professionals an earlier opportunity to identify and treat high risk groups. We found that certain baseline characteristics of critically ill patients with 2009 influenza A (H1N1) may be associated with increased ICU admission, including elevated CRP, leukocytosis and more abnormalities in chest radiography. Critically ill patients had severe acute lung injury which may explain these abnormalities in laboratory and radiographic findings. All patients had an influenza-like illness at presentation. None of the ICU patients received Oseltamivir before admission. According to the WHO guidelines on the pharmacologic management of influenza virus, patients who are at risk for pneumonia should be treated with Oseltamivir or Zanamivir as soon as symptoms develop, if possible [19]. At present, however, the quality of the evidence supporting such recommendations is low.

## Limitations

Our study has some limitations. First, patients who became infected in their community and did not go to the hospital were not included in our study. Second, the cases of infection in our patients are not clinically comparable with those in hospitalized patients in other countries because of differences in hospitalization practices. Finally, our data do not reflect the entire country but were collected in one university hospital at a representative city of Morocco.

## Conclusion

Close observation of patients infected with the 2009 pandemic influenza A (H1N1) virus infection provided us with several information. The influenza A (H1N1) virus infection affected young people particularly, with comorbid respiratory disorders. Risk factors with ICU admission were older age, long duration of symptoms, asthma, obesity, abnormalities of chest radiography, leukocytosis and higher CRP. Clinicians should be aware of complications of influenza A (H1N1) virus infection, particularly in patients with risk factors.

## Competing interests

The authors declare that they have no competing interests.

## Authors' contributions

LM draft the manuscript, BJ participated in the drafting of the manuscript. MC, AA, EFMT, ZAA, AK, FD, ESE, TR, BJ, ZA, SY, and CAC participated in the acquisition of data. AR conceived of the study, participated in the design of the study, performed the statistical analysis and interpretation of data, and gave the final approval of the manuscript. All authors read and approved the final manuscript. All authors read and approved the final manuscript

## References

[B1] Swine-origin influenza A (H1N1) virus infections in a school - New York City, April 2009 MMWR Morb Mortal Wkly Rep 2009 58 Dispatch 1 3 http://www.cdc.gov/mmwr/preview/mmwrhtml/mm58d0430a1.htm 19444151

[B2] Swine influenza A (H1N1) infection in tow children- Southern California, March-April 2009 MMWR Morb Mortal Wkly Rep 2009 58 400 2 http://www.cdc.gov/mmwr/preview/mmwrhtml/mm5815a5.htm 19390508

[B3] Update: infections with swine-origin influenza A (H1N1) virus-United States and other countries, April 28, 2009 MMWR Morb Mortal Wkly Rep 2009 58 431 3 http://www.cdc.gov/mmwr/preview/mmwrhtml/mm5816a1.htm 19407737

[B4] Naffakh N Van der Werf S April 2009: an outbreak of swine-origin influenza A (H1N1) virus with evidence for human-to-human transmission Microbes Infect 2009 11 725 8 10.1016/j.micinf.2009.05.002 19442755

[B5] Kent JN Lea CS Fang X Novick LF Morgan J Seasonal influenza vaccination coverage among local health department personnel in north Carolina, 2007-2008 Am J Prev Med 2010 39 74 7 10.1016/j.amepre.2010.03.007 20537842

[B6] Novel Swine-origin Influenza A (H1N1) Virus Investigation Team Dawood FS Jain S Finelli L Shaw MW Lindstrom S Garten RJ Gubareva LV Xu X Bridges CB Uyeki TM Emergence of a novel swine-origin influenza A (H1N1) virus in humans N Engl J Med 2009 360 2605 15 10.1056/NEJMoa0903810 19423869

[B7] Trifonov V Khiabanian H Greenbaum B Rabadan R The origin of the recent swine influenza A (H1N1) virus infecting humans Euro Surveill 2009 14 17 http://www.eurosurveillance.org/ViewArticle.aspx?ArticleId=19193 pii=19193 19422769

[B8] Update: drug susceptibility of swine origin influenza A (H1N1) viruses, April 2009 MMWR Morb Mortal Wkly Rep 2009 58 Dispatch 1 3 http://www.cdc.gov/mmwr/preview/mmwrhtml/mm58d0428a1.htm 19407738

[B9] Cao B Li XW Mao Y Wang J Lu HZ Chen YS Liang ZA Liang L Zhang SJ Zhang B Gu L Lu LH Wang DY Wang C Clinical features of the initial cases of 2009 pandemic influenza A (H1N1) virus infection in China N Engl J Med 2009 361 2507 17 10.1056/NEJMoa0906612 20007555

[B10] Deaths and hospitalizations related to 2009 pandemic influenza A (H1N1) - Greece, May 2009-February 2010 MMWR Morb Mortal Wkly Rep 2010 59 682 6 http://www.cdc.gov/mmwr/preview/mmwrhtml/mm5922a2.htm 20535092

[B11] World Health Organization Country Cooperation Strategy for WHO and Morocco 2004-2007 http://www.who.int/countries/en/cooperation_strategy_mar_en.pdf

[B12] New influenza A (H1N1) virus: global epidemiological situation, June 2009 Epidemiol Rec 2009 84 249 57 Wkly 19537358

[B13] Human infection with new influenza A (H1N1) virus: clinical observations from Mexico and other affected countries, May 2009 Wkly Epidemiol Rec 2009 84 185 9 19462531

[B14] Human infection with new influenza A (H1N1) virus: clinical observations from a school-associated outbreak in Kobe, Japan, May 2009 Wkly Epidemiol Rec 2009 84 237 44 19522091

[B15] Hospitalized patients with novel influenza A (H1N1) virus infection - California, April-May, 2009 MMWR Morb Mortal Wkly Rep 2009 58 Early Release 1 5 http://www.cdc.gov/mmwr/preview/mmwrhtml/mm58e0518a1.htm 19478723

[B16] Nichols JE Niles JA Boberts NJ Jr Human lymphocyte apoptosis exposure in influenza A virus J Virol 2001 75 5921 9 10.1128/JVI.73.13.5921-5929.2001 11390593 PMC114307

[B17] Domínguez-Cherit G Lapinsky SE Macias AE Pinto R Espinosa-Perez L de la Torre A Poblano-Morales M Baltazar-Torres JA Bautista E Martinez A Martinez MA Rivero E Valdez R Ruiz-Palacios G Hernández M Stewart TE Fowler RA Critically ill patients with 2009 influenza A (H1N1) in Mexico JAMA 2009 320 1880 7 10.1001/jama.2009.1536 19822626

[B18] Jain S Kamimoto L Bramley AM Schmitz AM Benoit SR Louie J Sugerman DE Druckenmiller JK Ritger KA Chugh R Jasuja S Deutscher M Chen S Walker JD Duchin JS Lett S Soliva S Wells EV Swerdlow D Uyeki TM Fiore AE Olsen SJ Fry AM Bridges CB Finelli L Hospitalized patients with 2009 H1N1 influenza in the United States, April-June 2009 N Engl J Med 2009 361 1935 44 10.1056/NEJMoa0906695 19815859

[B19] Who guidelines for pharmacological management of pandemic (H1N1) 2009 influenza and other influenza viruses http://www.who.int/csr/resources/publications/swineflu/h1n1_guidelines_pharmaceutical_mngt.pdf 23741777

